# Retraction: Effects of Tai Chi on Cognitive Function in Older Adults With Type 2 Diabetes Mellitus: Randomized Controlled Trial Using Wearable Devices in a Mobile Health Model

**DOI:** 10.2196/88646

**Published:** 2025-12-04

**Authors:** JMIR Editorial Office

**Affiliations:** 1 JMIR Publications Toronto, ON

The JMIR Publications Editorial Office is retracting the article “Effects of Tai Chi on Cognitive Function in Older Adults With Type 2 Diabetes Mellitus: Randomized Controlled Trial Using Wearable Devices in a Mobile Health Model” [1].

Following publication of this article, concerns were noted regarding the trial registration and institutional review board (IRB) approval number provided. The concerns identified were as follows:

- The cited trial registration number ChiCTR2200057863 is affiliated with a study conducted by individuals affiliated with the Shanghai University of Sport. None of the authors associated with this article are affiliated with this institution.
- The IRB number provided within the article “IRB-AF37-V1.0” does not appear to match the IRB approval reference within the trial registration “102772022RT029.”
- The study protocol listed within the trial registration significantly differs from the study described within the article. This includes substantial changes to participant sample sizes, inclusion/exclusion criteria, and outcome measures investigated following the intervention.
- Correspondence with relevant individuals at the Shanghai University of Sport identified that ChiCTR2200057863 was unrelated to the study published in the *Journal of Medical Internet Research*.

In response to the concerns, the corresponding author provided documentation of the ethical approval form “IRB-AF37-V1.0” and justifications for the misalignment with the protocol identified above. The response indicated that this study was initially carried out as a collaboration between the Shanghai University of Sport and the authors’ institution. The response also indicated that the trial applicants who do not appear as authors only secured trial registration sources and did not participate in the study. The corresponding author also indicated that the ethical approval for this study was rereviewed by the authors’ institute, resulting in a secondary IRB approval. The authors also stated that a formal amendment to the IRB approval was not necessary due to the core objectives pertaining to diabetes research remaining unchanged.

Following review of these responses, the Editorial Office determined that these responses did not adequately resolve the concerns. The authors were unable to provide a copy of the IRB approval from the Shanghai University of Sport listed in the trial registration. Furthermore, the Editorial Office determined that the significant changes in the study protocol from the original registration were not adequately justified. No evidence was supplied to justify the exclusion of the involvement and acknowledgement of individuals affiliated with the Shanghai University of Sport. Finally, representatives from the Shanghai University of Sport indicated that the trial registration ChiCTR2200057863 had no relation to the *Journal of Medical Internet Research* publication.

In light of concerns that cannot be resolved, the JMIR Publications Editorial Office retracts the article.

All authors agree with retraction.

## Abbreviations

IRB: institutional review board
